# Housing type and risk of depression – the mediating effects of perceived indoor annoyances and loneliness: a Danish cohort study, 2000–2018

**DOI:** 10.1186/s12889-025-22473-1

**Published:** 2025-04-11

**Authors:** Anne Marie Kirkegaard, Stine Kloster, Michael Davidsen, Anne Illemann Christensen, Klaus Martiny, Carlo Volf, Steffen Loft, Niss Skov Nielsen, Lars Gunnarsen, Annette Kjær Ersbøll

**Affiliations:** 1 Department of the Built Environment, Aalborg University, A.C. Meyers Vaenge 15, 2450 Copenhagen, SV Denmark; 2 National Institute of Public Health, University of Southern Denmark, Studiestraede 6, 1455 Copenhagen K, Denmark; 3 New Interventions in Depression, Mental Health Centre Copenhagen, Edel Sauntes Allé 10, 2100, Copenhagen Ø, Denmark; 4 Department of Clinical Medicine, University of Copenhagen, Blegdamsvej 3B, 2200, Copenhagen N, Denmark; 5 Department of Public Health University of Copenhagen, Section of Environmental Health, Øster Farimagsgade 5, 1014, Copenhagen K, Denmark; 6 Centre for Health Research, Zealand University Hospital, Strandboulevarden 64, 4800 Nykoebing F., Denmark

**Keywords:** Housing type, Housing tenure, Built environment, Indoor environment, Perceived loneliness, Perceived annoyance, Depression, Epidemiology, Cohort study, Mediation

## Abstract

**Background:**

Few studies have found that housing types and tenure might be associated with decreased mental health. Therefore, the aim was to investigate the association between housing type and the development of incident depression. Furthermore, quantifying the mediated effects through perceived indoor annoyances and perceived loneliness for the association between housing type and depression.

**Methods:**

In this cohort study, we followed 14,387 individuals. Data on depression, housing type, perceived indoor annoyances, perceived loneliness and several covariates were obtained from the Danish National Patient Register, the Danish National Prescription Registry, the Building and Housing Register, and the Danish Health and Morbidity Survey. The association between housing type and depression was estimated by using a generalised linear model with Poisson distribution of the number of incident depressions and a logarithmic transformation of risk time as offset. Causal mediation analysis estimated the total effect mediated by perceived indoor annoyances and perceived loneliness.

**Results:**

Individuals living in owned terrace houses, rented terrace houses, and rented apartments had a significantly higher incidence rate (IR) of depression compared to individuals in owner-occupied detached houses. Living in a rented apartment compared to owning a detached house was associated with an adjusted IRR for depression of 1.32 (95% CI 1.14, 1.53). Of this association, 11% could be attributed to perceived indoor annoyances and 8% to perceived loneliness. For individuals living in rented terrace houses compared to owner-occupied detached houses, perceived indoor annoyances mediated 6% of the association between housing type and depression.

**Conclusion:**

Individuals living in certain housing types had a significantly higher IR of depression compared to individuals in owner-occupied detached houses. Our findings suggest that some of the excessed depression incidents among individuals living in rented apartments and rented terrace houses compared to detached houses could be attributed to differences in the number of perceived indoor annoyances and perceived loneliness. However, future studies are needed to confirm the findings of the present study and address other pathways and possible causations.

**Supplementary Information:**

The online version contains supplementary material available at 10.1186/s12889-025-22473-1.

## Background

Depression is a common disease among people in high-income countries [1, 2] and have a high impact on general functioning [3, 4] with social and occupational impairment [5, 6]. In high-income countries, people spend 90% of their time in indoor spaces and approximately 65% indoors at home [7–9]. As depression is known to be more frequent in people living in apartments and in high-rise housings [7, 10], it makes it interesting to get a better understanding of selected risk factors that may mediate the effect of housing type on depression*.* This may strengthen strategies toward increasing mental health as a better understanding of potential pathways between housing type, and the risk of developing depression is crucial for targeted interventions.

Despite well-established research on depression, only a few authors have examined the relationship between housing type and depression, and with inconsistent findings [11–18]. For instance, a cross-sectional study of a random sample of Scottish adults (*n* = 2,828) found that housing type was not a significant predictor of depression, and that social renters were more likely than owners to be depressed [16]. In contrast, a longitudinal study of adults aged 40 and 60 (*n* = 691) from socially contrasting neighbourhoods in Glasgow, Scotland, concluded that housing type was significantly associated with depression and that housing tenure was not significantly associated with depression [14]. Another three studies showed mixed findings among elderly individuals living in their own homes and in assisted living facilities [12, 13, 15]. Others have found that individuals living in rented apartments, condominium residents, dorms or others, e.g. living as lodgers, have a significantly increased risk of poor psychological well-being and frequency of recent days with poor mental health compared to individuals living in private houses [19]. However, previous research has some methodological issues as they rely mostly on cross-sectional study designs; furthermore, they often lack control for confounding variables and had an elderly population.

Housing type and tenure are frequently used as proxies for socioeconomic status in studies of health inequalities [19, 20]. Socioeconomic factors may also impact the individuals’ housing type and tenure choice. Income or wealth enables individuals to buy homes, and owned homes tend to have higher daylight levels, less noise, damp, and other hazards, and thereby less stressful. Thus, housing tenure may affect health through housing stressors and types of area [14]. Even within social classes, there are strong relations between rental housing tenure and higher death rates [21–23]. Whether this is a result of personal differences between owners and renters, or of differences between housing, or both, remains uncertain [16].

According to current research, housing enforces different psychological, physical and social conditions on residents that may have several pathways to impaired health [16, 19, 24]. Studies have consistently demonstrated an association between loneliness and depression [25]. Furthermore, it has been found that housing types are associated with loneliness. People living in apartments tend to be more socially isolated and lonelier [7], which plausibly may affect the risk of depression for this group. Housing type may also be associated with perceived indoor annoyance [26]. Perceived indoor annoyances are generally not considered a risk factor for depression, although previous research has shown that these may be risk factors [11, 13–15, 17, 18]. Several mechanisms have been proposed to explain why perceived annoyances are expected to affect depression. For instance, annoyances might be considered a proxy for dissatisfaction and distress associated with actual exposure [27–32]. In addition to causing annoyance, environmental factors, e.g., air pollution, could also potentially lead to depression through biological pathways [33–35]. Furthermore, annoyances might also alter the annoyed individual's behaviour, such as reducing physical or social activities, potentially leading to mental health issues [34]. However, adverse effects of perceived indoor annoyances or perceived loneliness have not yet been documented for the relation between housing type and depression. Hence, the present study appears to be the first to investigate the mediated effect of perceived indoor annoyances and perceived loneliness on the association between housing type and depression.

The aim of this study was to investigate the association of housing type with the development of incident depression among Danish residents. Furthermore, to quantify the mediated effects through the number of perceived indoor annoyances and perceived loneliness.

## Methods

### Study design

This is a register-based observational cohort study with a closed cohort, where the study population was defined based on a cross-sectional study. Eligible individuals were included from the Danish Health and Morbidity Survey in the year 2000 (DHMS 2000), and linked to data from various Danish registers, thus enabling individuals to be followed for several years to identify health and social conditions. DHMS 2000 was a cross-sectional survey collecting self-reported data on health, social life and other living conditions [36]. All Danish residents have a unique personal identification number [37] that enables individual-level data linkage from different Danish registers, e.g., to obtain information from before cohort entry and up to December 31, 2018. This study investigates a combined exposure variable of housing type and tenure on the incidence of depression.

### Study setting and population

The DHMS 2000 enrolled Danish residents aged ≥ 16 years based on a simple random sampling design [36]. All invited individuals received an introduction letter that briefly described the purpose and content and emphasised that participation was voluntary [38]. A total of 22,486 individuals were invited, of whom 16,688 (74%) agreed to participate [36]. The design of the DHMS had several precautions to ensure that the sample was representative, which was confirmed in post hoc analyses (see details in [36]). Baseline data were collected in rounds during February, May, and September 2000 to limit the seasonal variation and were collected via personal interviews in the participants’ homes [36]. The interview covered questions regarding health behaviour, habits, lifestyle, social network, and working and living conditions [36]. Following the interview, participants completed a self-administered questionnaire, which mainly aimed to supplement the interview with more sensitive questions [38]. For example, the items used to determine mental health status.

The inclusion criterion was no prior history of depression (see definition in the next section). The exclusion criteria for this study were individuals who had missing information on housing type or tenure.

### Depression

Incident depression was defined as the first-ever hospital contact or redeemed antidepression medication. Information was retrieved from the Danish National Patient Register and the Danish National Prescription Registry [39, 40]. The Danish National Patient Registry holds individual-level data on every psychiatric admission since 1969 [41], somatic inpatients since 1977 [39], and from 1995 information on all outpatient activities, emergency room contacts, and activities in psychiatric wards [39, 41]. The Danish National Prescription Registry contains information on all prescriptions filled at community pharmacies in Denmark since 1995 [40].

Hospital contacts due to depression included primary, secondary, and additional diagnoses of the International Classification of Diseases (ICD) codes 8: 296.0, 296.2, 296.8, 298, 300.4, 311, and 313.1 until the 1 st of January 1994, and since then ICD- 10 codes: F32 and F33 (including subcategories). Antidepressant medication included Anatomical Therapeutic Chemical (ATC) code N06 A (including subcategories) except N06 AX12.

### Housing type

In Denmark, housing type and housing tenure are highly correlated; as of all detached single-family houses, 89% are owner-occupied, and 11% are occupied by tenants [42]. In contrast, of all apartments 12% is owner-occupied and 88% occupied by tenants. Less distinctive are the differences between semi-detached and terrace houses, as 32% are owner-occupied and 68% are occupied by tenants [42]. To gain more detailed knowledge of the effect of housing, the housing type exposure in the current study includes information on both housing type and tenure, as done similarly in previous research (e.g., [19, 43]).

**Fig. 1:**
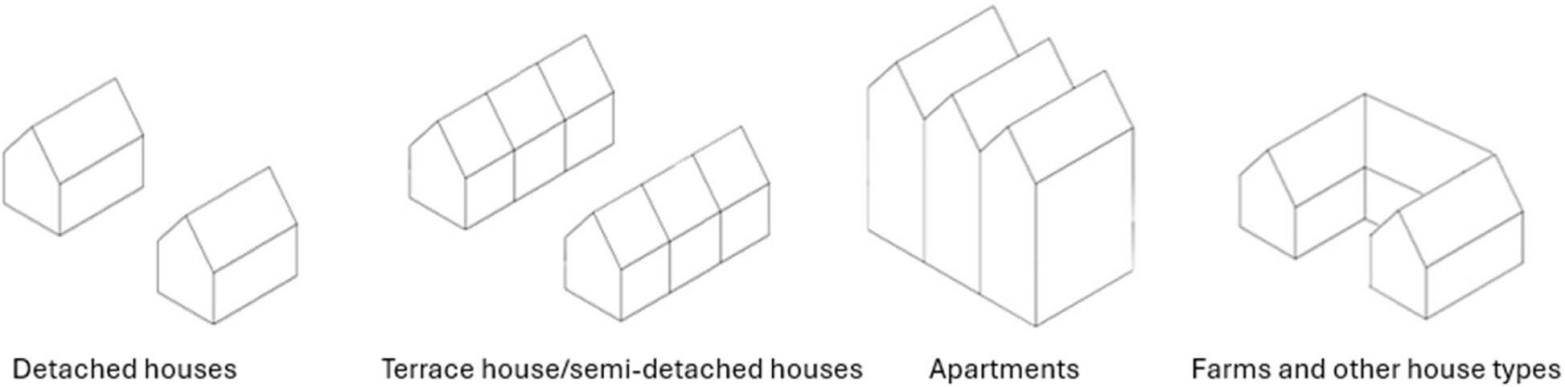
Illustration of housing types created by the authors. Each may be rented or owned

In this study, housing type consisted of seven categories: 1) Owned detached houses, 2) Owned terrace houses, 3) Rented terrace houses, 4) Rented apartments, 5) Owned farms, 6) Owned apartments and other housing types, and 7) Rented detached houses, farms, and other housing types (see Fig. 1). The latter categories have mixed housing types due to the sample size.

Housing tenure describes whether the residence was occupied by its owner or a tenant, and information was obtained from the Building and Housing Register [44]. The interviewers from the DHMS 2000 reported the housing type. The terrace house category included semi-detached houses, two-, three, or four-family houses, and chain, courtyard, and terrace houses. The category of other housing types concerned 3% of the study population and included houses the interviewer had classified as residential institutions, unknown or other (e.g., retirement homes or dormitories).

Of the enrolled individuals without prior depression, 4.5% had missing data on housing tenure and 0.6% on housing type [45], thus they were excluded based on the exclusion criteria.

### Perceived indoor annoyances and perceived loneliness

The mediators of interest were the number of perceived indoor annoyances and perceived loneliness obtained from the DHMS 2000 interview.

The number of perceived indoor annoyances was a continuous variable based on the number of self-reported annoyances. It was possible to report up to twelve annoyances which formed four categories: thermal discomfort, light levels, odour/stuffy air, and noise in the indoor environment at home. Participants reported which conditions they had been annoyed by within the last two weeks and whether they were slightly or very annoyed (see [26, 46, 47]). We categorised the answers for each item as not annoyed or annoyed and summarised the number of perceived indoor annoyances. Thereby, the number of perceived indoor annoyances could range between 0 and 12 annoyances for each individual in the main analysis.

Perceived loneliness was measured by one single-item question: “Does it ever occur that you are alone although you prefer to be together with other people?” with five response alternatives: “Yes, frequently”, “Yes, occasionally”, “Yes, rarely”, “No”, “Do not know” and not informed. The latter two were characterised as missing when analysed. Those individuals who answered “*yes, frequently*” were categorised as lonely.

### Covariates

Potential confounders were identified based on a Directed Acyclic Graph (DAG) using the DAGitty web application [48]. According to the DAG, one set of minimum sufficient adjustments was applicable: Age, cohabitation status, socioeconomic status (SES), urbanisation, and year of housing construction (see Supplemental Fig. 1). In addition, the analyses were adjusted for number of years lived in the residence at baseline and calendar year. We adjusted for the number of years lived in the residence to account for the accumulated risk of disease derived from the home as well as to account for the fact that individuals who are satisfied with their home also tend to live there longer. We adjusted for the calendar year since the incidence rate (IR) of depression varied during the study period [49]. The calendar year was categorised as 2000–2002, 2003–2005, 2006–2008, 2009–2011, 2012–2014, and 2015–2018.

Date of birth was obtained from the Danish Civil Registration System [50]. Age was categorised into 5-year intervals. Information on cohabitation status was obtained from the Danish Civil Registration System [50], but in case of missing, it was obtained from the DHMS 2000 [36]. Cohabitation status was defined as married/cohabiting (married, registered partnership or cohabitation) and living alone (separated, divorced, widowed, unmarried, or no cohabitation). As a proxy for socioeconomic status, we chose to adjust for the highest attainted educational level, as it captures long-term social status [51]. The highest attainted educational level was obtained from the Population’s Education Register with yearly updates [52]. We categorised educational level according to the International Standard Classification of Education System (ISCED) [53] and aggregated it into three groups: Mandatory (ISCED level 1–2), secondary or vocational (ISCED level 3–4), and medium or long education (ISCED level 5–8). Age, cohabitation status, and educational level were updated yearly when included in the analyses.

The Building and Housing Register obtained information on urbanisation (rural areas with less than 200 residents, towns with 200–4,999 residents, 5,000–49,999 residents, or ≥ 50,000 residents, missing), year of house construction (< 1960, 1960–1978, or ≥ 1979), as well as individuals’ change of address from which we calculated the number of years lived in the house at baseline (in quartiles: < 2.43, 2.43–7.65, 7.66–18.37, and ≥ 18.37 years) [44].

From the DHMS 2000, we furthermore obtained information to describe the population in more detail and to include in sensitivity analyses. This included information on self-rated health (very good, good, fair, poor, very poor), smoking status (smoker, non-smoker), season of enrolment, and mental health status [36]. Mental health status was based on the 36-item Short-Form Mental Health (SF- 36 MH) scale, which was a part of the DHMS 2000 questionnaire. The SF- 36 MH scale was a continuous variable ranging from 5 to 30 points (a few points represented a low mental health score) based on five self-reported items. A random sample of 10,458 individuals received the SF- 36 MH scale items [38, 54], of which 8,663 individuals were included in the current study. Further variables obtained from the Danish Civil Registration System and the Building and Housing Register include sex (male, female), ethnicity (Danish, non-Danish), employment status (not in the workforce, unemployed, receive one’s education, wage earner, co-operating spouse, and independent business owner), family equivalent income (in quantiles, assessed yearly), number of residents (1, 2, 3, 4, ≥ 5), and area per person (< 40, 40–79, ≥ 80 square metres per resident) [44, 50].

### Risk time

Risk time for each individual was calculated as the time from the date of the interview or six months after moving into the house where the survey was answered, whichever occurred last. The cut-off at six months was applied to ensure depressions were linked to the home of interest [55, 56], thus six months was assessed as the minimum latent period. Individual risk time continued until the first incident of depression, death, emigration, six months after moving to another address, or the end of the study on December 31, 2018, whichever came first.

### Statistical analyses

For descriptive purposes, we calculated percentages and mean with standard deviations (SDs) for the baseline characteristics, number of incident depression events, person-years (PY) at risk, and IR per 10,000 PY.

The association between housing type and depression was estimated by using a generalised linear model with Poisson distribution of the number of incident depressions and a logarithmic transformation of risk time as offset [57]. The model assumption required that the depression IR was constant within each time interval, thus we split risk time by age and calendar year using *SplitMulti* from the PopEpi package after having constructed a Lexis object using the Epi package in R [58]. Incidence rate ratios (IRRs) were estimated with 95% confidence intervals (CIs) in crude and adjusted models with owned detached houses as reference group. To take account of non-response, the models included weights computed by Statistics Denmark based on e.g., sex, age and income [59, 60].

**Fig. 2:**
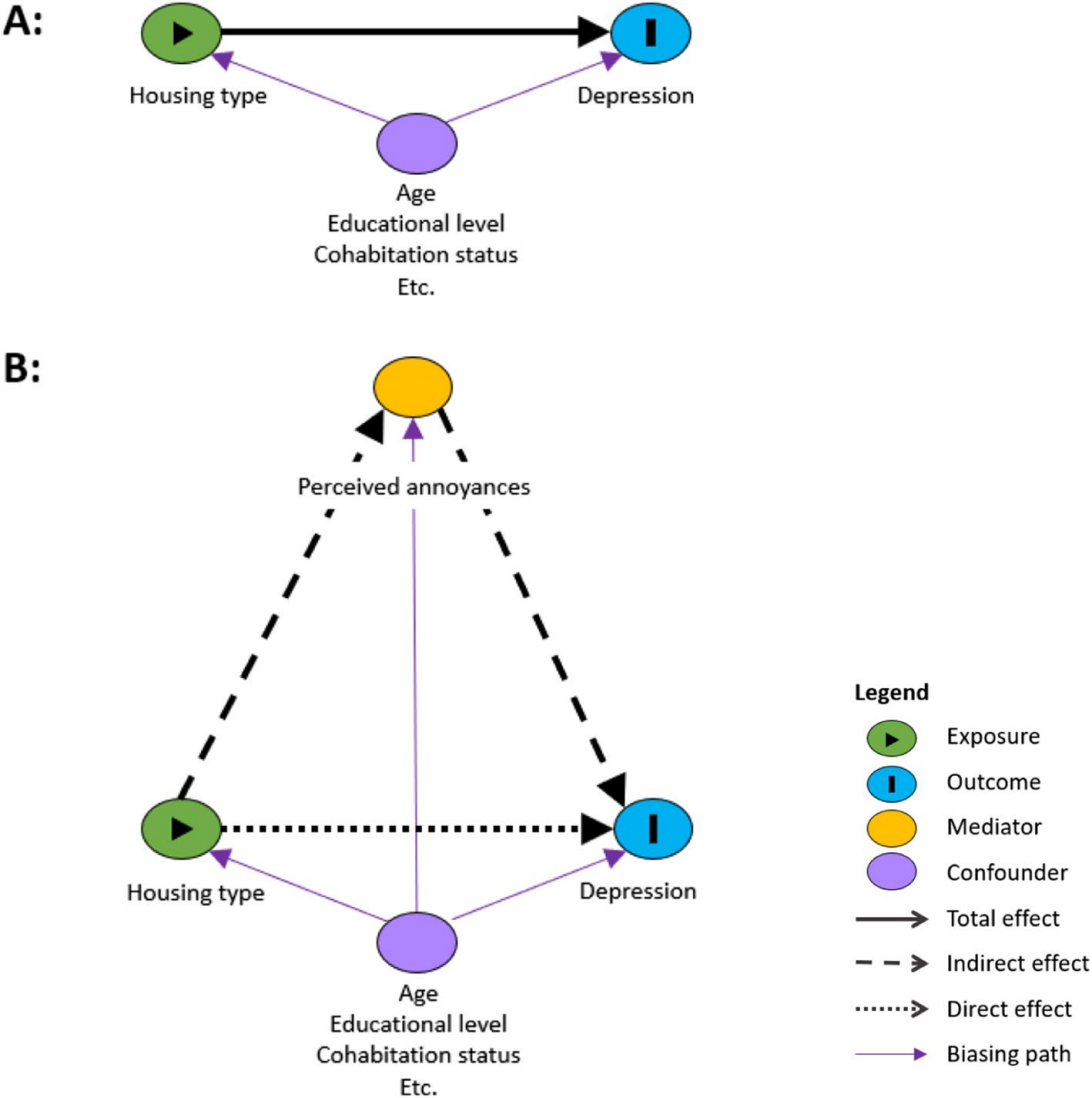
Illustrative example of DAG for the total effect (**A**) and the mediated/indirect effect (**B**)

We conducted causal mediation analyses to estimate the potential mediation effects of perceived indoor annoyances and perceived loneliness on the association between housing type and incident depression. See Fig. 2 for our hypothesised mediation pathway.

The mediation analysis required a dichotomous exposure; thus, owned detached house was set as the reference group. Each housing type that showed significance in the main analysis was compared with owned detached houses in separate mediation analyses. This resulted in the following analyses for each potential mediator: Rented apartment vs. owned detached house, rented semidetached terrace house vs. owned detached house, and owned semidetached terrace house vs. owned detached house.

The causal mediation analysis was performed to estimate the total effect (TE) of housing type on incident depression, the natural direct effect (NDE) of housing type on incident depression controlling for the mediator, and the natural indirect effect (NIE) of housing type through the mediators on incident depression. Finally, the proportion of the total effect mediated (PM) was estimated and expressed as a percentage and reported with 95% CI. The sum of NDE and NIE is the TE [61]. The NDE can be interpreted as the IRR of depression among individuals living in one housing type (e.g., apartments) versus individuals living in owned detached houses as reference when controlling for perceived annoyances (i.e., comparing the two housing types for individuals with the same level of the mediator). The NIE represents the effect of housing type on the incidence rate of depression through perceived annoyances. The NIE can be interpreted as the IRR of depression when the mediator (i.e., perceived annoyances or loneliness) is changed corresponding to the difference between the two housing types compared. The PM can be interpreted as the change in the effect of housing type on incident depression due to mediation by perceived annoyances or perceived loneliness relative to the TE.

The statistical test of mediation was based on two models, one model examining the association between housing type as exposure and incident depression as the dependent variable controlling for the mediator and one model examining the association between housing type as exposure and the mediator as the dependent variable. Both models were adjusted for age, cohabitation status, SES, urbanisation, year of housing construction, number of years lived in the residence at baseline and calendar year and weighted for non-response. In the first model of the association between housing type and incident depression we applied a generalised linear model with a Poisson distribution of the number of events as outcome and a logarithmic transformation of risk time as the offset value [57]. Secondly, a model of the association between housing type and the mediator was used. As perceived indoor annoyance was a discrete variable, the housing type-mediation association was estimated by a Poisson regression of the number of perceived indoor annoyances. Perceived loneliness was a binary variable; thus, we employed a logistic regression for this housing type-mediation association.

The mediation analyses used Quasi-Bayesian confidence intervals with 1,000 simulations and robust standard error. The mediation analysis was performed using the *mediate* function from the R package *mediation* [61].

All analyses were performed in R version 4.4.1.

### Supplemental analyses

To assess the robustness of the results, we repeated the association and mediation analyses with few adjustments. First, we repeated the mediation analyses for perceived indoor annoyances using the summarised number of annoyances within each category of annoyances, i.e., noise, thermal discomfort, low levels of light, and odour/stuffy air. Thereby, examining whether one of the categories was primary in the effects seen in the main analyses.

Secondly, we excluded the 9% of individuals with the lowest SF- 36 MH score to test whether the findings changed after excluding individuals with potentially undiagnosed depression [62]. Thirdly, we excluded individuals with poor, very poor and missing self-rated health, as poor self-rated health might also be correlated with an undiagnosed depression [63, 64]. Fourthly, we excluded individuals under 30 years old to test whether the educational level was an appropriate proxy for socioeconomic status. Furthermore, we tested employment status (in three and six groups respectively), family equivalent income (in quantiles), and employment and educational level simultaneously as proxies for socioeconomic status. The fifth supplemental analysis included a cross tabulation and chi-squared test of the number of perceived indoor annoyances and perceived loneliness to test the independence of the two mediation variables.

### Missing data

The number of missing data was low on mediators as for perceived indoor annoyances 16 individuals had missing data and for perceived loneliness 41 individuals. The only confounding variables with missing data were year of construction with ≤ 3 individuals and educational level with 415 individuals (3%). The SF- 36 MH scale was missing for 5,724 individuals (40%), since only a subpopulation received the questions. The rest of the covariates used for descriptive purposes were missing for between 0 and 18 individuals.

## Results

**Fig. 3:**
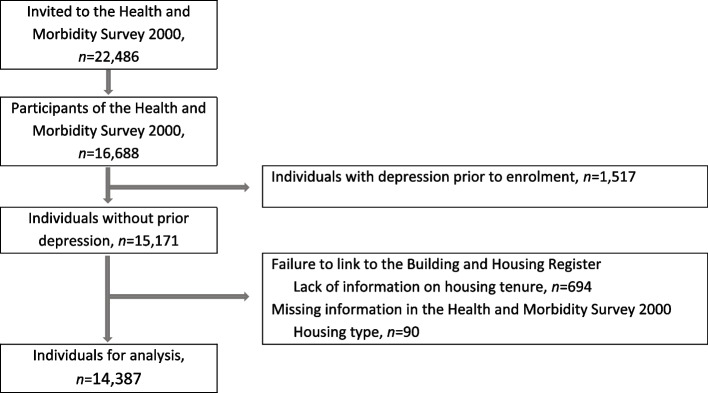
Flow diagram of individuals in the study

The study population consisted of 14,387 individuals without prior depression and available information on housing type and tenure (see Fig. 3 for details of the flow chart of individuals).

Most individuals lived in owned detached house (49%), while 7% and 9% lived in owned and rented terrace houses, respectively, 18% in rented apartments, 8% in owned farms, 4% in owned apartments and other housing types, and 5% in rented detached houses, farms and other housing types. Baseline characteristics of the study population stratified by housing type is shown in Table 1. The percentage of individuals who frequently perceived loneliness ranged from 1.8% in owner-occupied detached houses to 4.7% in rented apartments (Table 1). Individuals in apartments (both owners and tenants) were generally more likely to perceive indoor annoyances compared to individuals in detached houses (Table 1).

**Table 1:**
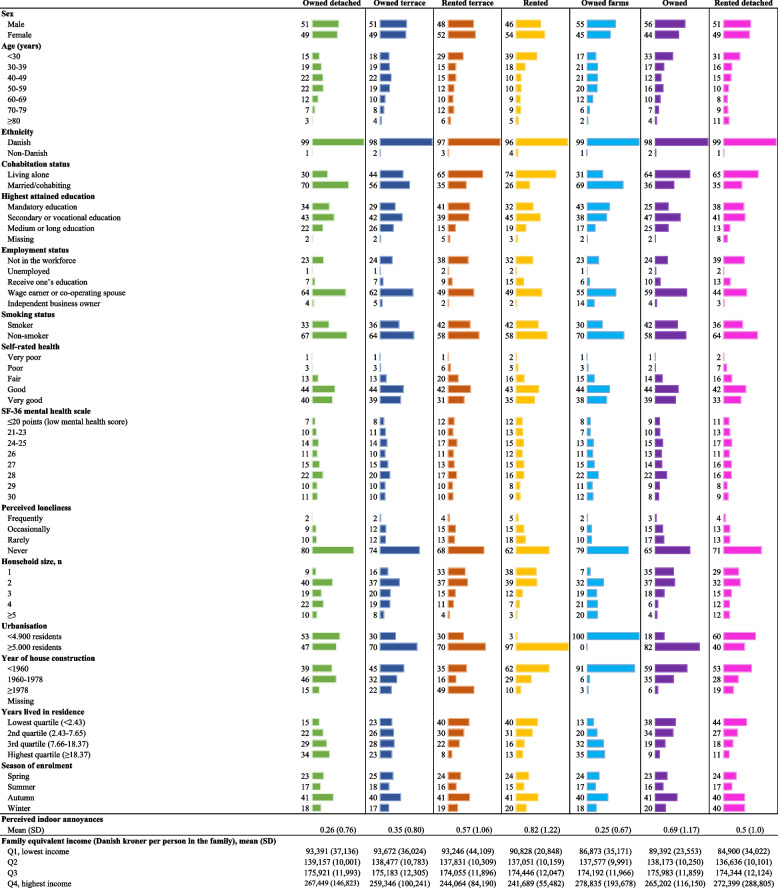
Baseline characteristics of the study population, % (*n*= 14,387)

Individuals were followed for an average of 9 years, during which 2,049 individuals had their first depression diagnosis. The crude absolute rate of depression was 158 per 10,000 PY at risk and ranged from 123 for owned apartments and other housing types to 239 for rented terrace houses (Table 2).

**Table 2:** Poisson regression of rates of depression among individuals (*n* = 14,387) from the Danish Health and Morbidity Survey 2000

**Housing type**	***N*** (14,387)	**Incident depression**				
		**Number of events** (2,047)	**PYs at risk** (129,488)	**IR per 10,000 PY** (158)	**IRR** ^a^** (95% CI)**	**Adjusted IRR**^a, b^ **(95% CI)**
Owned detached houses	7,114	1,080	77,774	139	1 (reference)	1 (reference)
Owned terrace houses	1,014	157	9,325	168	1.27 (1.08, 1.50)	1.23 (1.03, 1.45)
Rented terrace houses	1,344	195	8,149	239	1.75 (1.50, 2.03)	1.32 (1.11, 1.57)
Rented apartments	2,558	337	15,175	222	1.59 (1.42, 1.78)	1.32 (1.13, 1.52)
Owned farms	1,084	154	11,440	135	0.95 (0.78, 1.13)	0.89 (0.71, 1.12)
Owned apartments and other housing types	524	44	3,575	123	0.92 (0.69,1.20)	0.80 (0.58, 1.06)
Rented detached houses, farms and other housing types	749	80	4,048	198	1.54 (1.23, 1.91)	1.18 (0.92, 1.49)

*N* number of individuals, *PY* person-years, *IR* incidence rate, *IRR* incidence rate ratio, *CI* confidence interval

^a^Weighted for non-response

^b^Adjusted for age, cohabitation status, educational level, urbanisation, year of housing construction, number of years lived in the residence at baseline, and calendar year

Housing type was significantly associated with depression for owned and rented terrace houses with adjusted IRR of 1.23 (95% CI 1.03, 1.45) and 1.32 (95% CI 1.11, 1.57), respectively, and for rented apartments with an adjusted IRR of 1.32 (95% CI 1.14, 1.53) compared with detached houses. The other housing types had not significantly different depression incidences compared to owned detached houses (Table 2).

The mediation effects of perceived indoor annoyances and loneliness between housing type and depression are presented in Table 3. No indirect effect was seen in the comparison of incident depression between individuals living in an owned semidetached terrace house and individuals living in an owned detached house. The number of perceived indoor annoyances mediated 11% of the association between rented apartments and depression and 6% for rented terrace houses with owned detached houses as reference (Table 3). Moreover, 8% of the association between rented apartments and depression was mediated through perceived loneliness compared to detached houses (Table 3). In supplemental analyses, we found that 8% of the association between housing type and depression was mediated by noise among individuals living in rented apartments compared to owner-occupied detached houses. The analysis further showed thermal annoyances mediated 4%, annoyances due to low light levels mediated 5%, and odour annoyances mediated 3% for individuals living in rented apartments compared to owner-occupied detached houses (see Supplemental Table 1). In contrast, when comparing rented terrace houses with owned detached houses thermal annoyances mediated most of the effect to depression with 4% (see Supplemental Table 1).

**Table 3:** Association between housing type and incident depression mediated by the number of perceived indoor annoyances and perceived loneliness^a^

**Potential mediator**	**Adjusted IRR (95% CI)**^b^		
	Owned semidetached terrace house vs. owned detached house	Rented semidetached terrace houses vs. owned detached houses	Rented apartments vs. owned detached houses
**Number of perceived indoor annoyances**^**c**^			
Total effect	1.003 (1.0001, 1.01)	1.005 (1.001, 1.01)	1.01 (1.002, 1.01)
Natural direct effect	1.003 (1.0001, 1.01)	1.004 (1.001, 1.01)	1.00 (1.002, 1.01)
Natural indirect effect	1.00001 (0.99, 1.00004)	1.0003 (1.00004, 1.001)	1.00 (1.0003, 1.001)
Proportion mediated, % (95% CI)	0.3 (− 1.6, 2.9)	6.3 (0.9, 23.0)	10.8 (5.4, 24.4)
**Perceived loneliness**^**d**^			
Total effect	1.00 (1.00, 1.01)	^**e**^	1.01 (1.01, 1.02)
Natural direct effect	1.00 (1.00, 1.01)	^**e**^	1.01 (1.01, 1.01)
Natural indirect effect	1.00 (0.99, 1.00)	^**e**^	1.00 (1.0005, 1.002)
Proportion mediated, % (95% CI)	3.5 (− 2.7, 16.3)	^**e**^	8.0 (4.0, 14.6)

Owned detached houses were the reference group

*CI* confidence interval

^a^Mediated effects were only estimated for those housing types that showed significant associations in the main analysis

^b^Weighted for non-response

^c^Exposure-outcome association and exposure-mediator association were adjusted for age, cohabitation status, educational level, urbanisation, year of housing construction, number of years lived in the residence at baseline, and calendar year

^d^Exposure-outcome association and exposure-mediator association were adjusted for urbanisation and construction year. The other confounding variables were correlated with perceived loneliness

^**e**^The result could not be estimated

Excluding the individuals from the cohort with the lowest mental health score and poorest self-rated health, respectively, yielded no differences in the findings of the association between housing type and depression (see Supplemental Table 2 and Table 4). However, the mediated effect decreased for the supplemental analysis of mental health (giving insignificant associations), while the mediated effects slightly increased in the analyses of perceived annoyance in the population with fair and good self-rated health (see Supplemental Table 3 and Table 5). When individuals under 30 years old were excluded, the results did not change notably compared with the main analysis (see Supplemental Tables 6–7). Furthermore, the results did not change remarkably when the analyses instead of the educational level were adjusted for either of the following as proxies for socioeconomic status: Employment status, family equivalent income, and employment and educational level simultaneously (data not shown). Finally, a supplemental analysis showed that individuals who were lonely did not significantly differ from individuals who perceived many annoyances (see Supplemental Table 8).

## Discussion

In this large, national representative cohort of adults living in Denmark, we found a significant association between housing type and depression. The IR of depression was significantly higher among tenants in apartments, owners and tenants of terrace houses compared to the IR among individuals in owner-occupied detached houses. Our findings show that approximately 11% of the association between housing type and depression incidents among tenants in apartments was explained by perceived indoor annoyances when compared to individuals living in owned detached houses. Whereas, 8% of the association between housing type and depression incidents was explained by perceived loneliness when comparing tenants in apartments to owners of detached houses. With respect to tenants in terrace houses the findings indicated that approximately 6% of the association between housing type and depression incidents was explained by perceived indoor annoyances when comparing to individuals in owned detached houses.

The literature on the association between housing type and depression is limited. However, in line with the findings of this study, the majority of previous studies found that certain housing types were associated with depression [11, 13–15, 17, 18] while others found no association [12, 16]. A study found a significantly increased depression score among renters compared with owner-occupiers, as well as for individuals living in ‘four in a block’ and flats compared to houses [14]. Further, living at the ground level was found to impact mental health negatively as respondents living at ground level had the highest depression mean score, while the lowest were found for those living on floor level 4 and above [14]. In contrast, another study found that older individuals living in high-rise apartments experienced higher levels of depression compared to those living in community housing [15].

Several factors could explain the associations found in this study. It might be that the internal conditions and the quality of the house may be poorer in rented homes compared to owned ones. The quality of the indoor environment may if poor lead to worries about the household members'health, concerns and embarrassment of the homes’ physical appearance in such a way that it may lead to a general state of dissatisfaction, chronic stress, and development of depression [10, 14, 21].

The association might also be explained by factors related to the physical structure of housing, e.g., building materials and design [10, 21]. In a supplemental analysis, we observed that annoyances due to low light levels mediated most of the effect for the association between rented terrace houses and depression among the examined annoyance categories when compared to owned detached houses. Low light levels may be explained architecturally; terrace houses often only have direct access to daylight from two sides [65]. Also, the finding that noise annoyance was the most impactful mediator studied for individuals living in rented apartments compared to owned detached houses may be explained architecturally since noise between floors of apartments is a common and well-described phenomenon [66, 67]. Different housing types and built environments might also affect the behaviour of the occupants and thereby indirectly the health [19, 7].

Our findings indicate that tenants were more likely to develop depression compared to individuals in owned properties independently of the type of house. Individuals living in owned farms, and owned apartments and other housing types did not have significantly different risk of depression than individuals in owned detached houses. Although individuals in owned terrace houses had a significantly higher risk compared to individuals in owned detached houses, the risk for tenants in terrace houses was higher. Individuals in rented apartments also had a significantly higher IR compared to that of individuals living in owned apartments and other housing types.

Differences in housing tenure have been found related to inequalities in health, as owner-occupiers tend to be healthier than renters [16, 21]. The psychological impact of renting might also affect the mental health of tenants, as renting is more insecure than owning and seems to grant less autonomy and social status [20, 68]. However, owners that pay off a mortgage might also feel increased insecurity if they fall behind on payments [21] or not have savings for house repairs. A study by Connolly questioned whether tenure acts as socioeconomic status proxy by explaining income and wealth accumulated across the life course and facilitating home ownership or alternatively explaining other characteristics such as housing quality or location [20]. Ellaway and Macintyre suggested rather than housing tenure simply being a marker of income, it may be used to predict housing and neighbourhood conditions [14].

The location, the social and physical characteristics of the area surrounding the home may also play a role in the findings of a housing type–depression association. Owner-occupied houses tend to be located in open, more wealthy areas, while rental properties in more dense, deprived areas [14, 20]. Architecturally, this often has implications for access to daylight, sunlight and fresh air.

Area of residence may impact mental health through a number of potential ways, such as norms of behaviours, access to services, parks and sports facilities, and the potential stress associated with a deprived area that often experiences higher levels of ‘incivilities’ (e.g. vandalism, litter and graffiti) and fear of crime [10, 14, 20, 21]. Neighbourhood conditions may also directly influence habits of privacy, child-rearing, and housekeeping, which indirectly may affect health [21, 69]. Suggesting that housing type and tenure might provide information on exposures to health, promoting or damaging features of the residence and its near environment [17, 20].

Personal characteristics might also explain some of the observed associations between housing type and depression. Moreover, some individuals might be more susceptible to the negative effects of the built environment on mental health [10]. Hiscock et al. found age, self-esteem and income to be the most consistent factors in explaining the relation between tenure and health [16]. Park and Kim found evidence of different psychological reactions to cumulative exposure to poor housing conditions between age groups [70]. Young and middle-aged adults had increased levels of depressive symptoms in the first years of exposure to poor housing conditions but thereafter remained at a similar level, whereas older adults continued to develop greater depressive symptoms over time [70].

To our knowledge, no previous studies have examined perceived indoor annoyances or perceived loneliness as mediators for the housing type–depression relation. However, a Korean study found a mediating effect of loneliness between age-friendly environments and depressive symptoms among older adults above 65 years old [71]. The mediated effect was not observed for younger adults (18–44 years old), which illustrated different ways that environments were weighted by age groups and the importance of being fulfilled emotionally to reduce depressive symptoms [71]. Another study found that a sense of belonging partly mediated the association between housing type and depressive symptoms among older adults [13].

In the current study, we found that perceived indoor annoyances and perceived loneliness only partly mediated the association between housing type and depression for tenants in apartments and terrace houses and perceived loneliness also for owners of terrace houses compared with owners in detached houses. Since perceived indoor annoyances and perceived loneliness are known to increase the risk of depression [25, 32, 47, 72–74], it may indicate that the current study is consistent with previous findings. However, these pathways need to be confirmed in future studies, as well as other pathways need to be investigated to better understand the relation between housing type and depression.

The proportion mediated by perceived loneliness decreased from 8.0% (95% CI: 4.0, 14.6) in the full sample to 2.5% (95% CI: − 1.3, 9.6) after excluding individuals with the poorest mental health. This reduction in the mediation effect between housing type and depression may be explained by a stronger or more direct relationship between perceived loneliness and depression in individuals with poorest mental health, which could have amplified the mediation effect. When these individuals were excluded from the sample, the relationship between housing type and depression became less influenced by perceived loneliness, suggesting that the poorest mental health group may have been driving the observed effect in the full sample. This also implies that individuals with fair or good mental health might be more robust to the impact of loneliness on risk of depression.

The findings did not change substantially after excluding individuals with the poorest self-rated health, indicating that we most likely did not include individuals with undiagnosed depression at baseline. Thereby supporting the main findings.

Additionally, the findings from the full sample did not change after excluding individuals younger than 30 years old indicating that educational level was an appropriate proxy for socioeconomic status. This was also supported in the results of the analysis of the full sample when educational level was exchanged by employment status, family equivalent income, and employment and educational level simultaneously as proxies for socioeconomic status.

### Strengths and limitations

The main strengths of this study were the cohort design, large-size sample derived from a representative population of Danes, up to 19 years of follow-up, the linkage to population-based registers on disease, housing and demographic characteristics, and unique self-reported data. Furthermore, the high response rate of 74% and analyses that accounted for non-responses by applying statistical weights.

Several limitations should also be considered. There may have been reverse causality, as individuals more prone to depression might choose to live in lower-quality housing. However, since individuals included had no prior medical history of depression, we believe the impact on the findings is limited. The risk remains for individuals who enter the study with undiagnosed depression, but we consider the risk small, as risk time started six months after moving into the housing or at the interview date, whichever occurred last. The prospective cohort design and confounder adjustments addressed the direction and relationship between variables. In the mediation analyses, the exposure and mediators were based on cross-sectional data, i.e., measured at baseline. However, it is safe to assume that housing type comes before the measured mediators, as housing type was fixed at moving into the residence, thus limiting the risk of reverse causality.

Residual confounding presents another potential risk of bias in our results. To address this, we identified potential confounders through a review of the literature and a DAG to visualize the relationship between variables and adjusted the analyses accordingly. However, the possibility of bias from unknown confounders remains. In the analyses age was included in 5-years intervals and educational level in three groups, yet a finer graduation was possible, however, we do not consider that the risk of residual confounding had been minimized notably.

It is important to recognize that this study focuses on depression diagnoses and antidepressant prescriptions, which do not necessarily equate to depression. Many individuals do not seek a formal diagnosis or medication for their condition [75, 76]. Additionally, antidepressant prescriptions are not an ideal measure of depression, as these medications are also commonly prescribed for a variety of other health issues, such as back pain and anxiety [77, 78]. Depression diagnoses in the Danish National Patient Registry are considered of high quality and validated [41, 79]. While severe mental disorders are nearly fully registered; mild to moderate mental disorders are not completely recorded [41]. The data from the Danish National Prescription Registry are also deemed complete and valid due to the systems designed to reduce data entry errors and the financial incentives provided to pharmacies to ensure full registration of purchases [40]. We consider that only having prescriptions which were obtained from community pharmacies did not affect the findings considerably. Individuals not identified via prescriptions from hospitals or clinics were most likely also registered as having a hospital contact or would after the hospitalization redeem prescriptions at the community pharmacy, and thereby be identified, however this hypothesis is untested.

We risked including individuals with a prior depression since information was limited to psychiatric admissions and somatic inpatients before 1995 [39, 41]. However, research shows that individuals with five or more depression-free years are not at increased risk of recurrence [80, 81], so by having information for five years before study enrolment in year 2000, we should be able to detect individuals with prior depression due to the increased risk of recurrence. In case of any inclusion, the misclassification was non-differential.

### Implications for research and practice

Our findings indicate that individuals living in rented properties, such as tenants in apartments, were more likely to develop depression compared to individuals in owned properties. Furthermore, that the association between housing type and depression was partly mediated by perceived indoor annoyances and perceived loneliness. Based on these findings, we propose that mental health policies and those who manage rental properties focus on reducing sources of indoor annoyances such as noise, low light levels, low/high temperatures, and odours/stuffy air. Interventions, such as noise reduction in apartment buildings or improved access to daylight in rented properties, are examples of settings where indoor annoyances could be reduced, as our supplemental analysis showed that these mediated most of the annoyance effect between housing type and depression.

Moreover, it is essential to reduce perceived loneliness among tenants in apartments and terrace houses. For example, by implementing spaces and programs that aim to decrease social isolation among residents, and support neighbouring and informal contact with other residents [10]. A simple supplemental test showed a significant overlap between individuals who perceived loneliness and perceived indoor annoyances. One may argue that individuals who perceive loneliness might be more vulnerable to perceive indoor annoyances, thus, interventions for loneliness should be prioritized to prevent some cases of depression derived from living in rental properties.

Overall, future studies are needed to confirm the findings of the present study and to try to explain housing type differences in depression in order to reduce the risk of depression.

## Conclusions

Our findings showed that individuals living in owned terrace houses, rented terrace houses, and rented apartments had a significantly higher incidence rate of depression compared to individuals living in owned detached houses. No significant association was seen between individuals living in owned farms, owned apartments and other housing types, or rented detached houses, farms and other housing types and incidence rate of depression compared to individuals living in owned detached houses. We found that the higher incidence of depression among tenants in apartments was partly mediated through perceived indoor annoyances (11%) and perceived loneliness (8%). Furthermore, that perceived indoor annoyances mediated 6% of the association for individuals living in rented terrace houses. Thus, the differences in depression between housing types would in theory possibly be reduced if tenants could reduce their perception of indoor annoyances and the frequency of perceived loneliness corresponding to the levels of individuals in owned detached houses.

## Supplementary Information

Supplementary Material 1.

## Data Availability

The data that support the findings of this study are available from Statistics Denmark, but restrictions apply to the availability of these data, which were used under license for the current study, and so are not publicly available. Data access can however be granted to researchers in Danish research environments with a well-defined research project after approval from the Danish Data Protection Agency and Statistics Denmark.

## Abbreviations

ATC: Anatomical Therapeutic Chemical
CI: Confidence Interval
DAG: Directed Acyclic Graph
ICD- 8: The 8 th revision of International Classification of Diseases
ICD- 10: The 10 th revision of International Classification of Diseases
IR: Incidence rate
IRR: Incidence rate ratio
ISCED: The International Standard Classification of Education System
PY: Person-year
SES: Socioeconomic status
