# Supplementary material for: Housing type and risk of depression – the mediating effects of perceived indoor annoyances and loneliness: a Danish cohort study, 2000–2018
Source: BMC Public Health. 2025 Apr 11;25:1372. doi: 10.1186/s12889-025-22473-1 (PMC11987425; doi:10.1186/s12889-025-22473-1)
Supplement: Supplementary file 1 — Supplementary Material 1. [file 12889_2025_22473_MOESM1_ESM.pdf]

**Supplemental Material**

Kirkegaard AM*, Kloster S, Davidsen M, Christensen AI, Martiny K, Volf C, Loft S, Nielsen SN, Gunnarsen L, and Ersbøll AK. Housing type and risk of depression – the mediating effects of perceived indoor annoyances and loneliness: A Danish cohort study, 2000-2018. BMC Public health, 2025

* Author for correspondence: Anne Marie Kirkegaard, e-mail: amk@build.aau.dk

**Table of contents**

Supplemental Figure 1. Simplified Directed Acyclic Graph (DAG) highlighting variables of importance in the analysis of the association between housing type and depression.

Supplemental Table 1. Proportion mediated of housing type on depression for selected potential mediators [a]: number of perceived indoor annoyances due to either noise, thermal discomfort, low levels of light, or odour/stuffy air

Supplemental Table 2. Poisson regression of rates of depression among a sample of individuals with ≥21 points in the SF-36 mental health score at baseline (n=7,906) from the Danish Health and Morbidity Survey 2000

Supplemental Table 3. Proportion mediated of housing type on depression for selected potential mediators [a] when including number of perceived indoor annoyances as a possible mediator among a sample of individuals with ≥21 points in the SF-36 mental health score at baseline

Supplemental Table 4. Poisson regression of rates of depression among individuals with very good, good, and fair self-rated health at baseline (n=13,742) from the Danish Health and Morbidity Survey 2000

Supplemental Table 5. Proportion mediated of housing type on depression for selected potential mediators [a] among individuals with very good, good, and fair self-rated health at baseline

Supplemental Table 6. Poisson regression of rates of depression among a sample of individuals ≥30 years old (n=11,993) from the Danish Health and Morbidity Survey 2000

Supplemental Table 7. Proportion mediated of housing type on depression for selected potential mediators [a] among a sample of individuals ≥30 years old

Supplemental Table 8. Table of perceived indoor annoyances and perceived loneliness

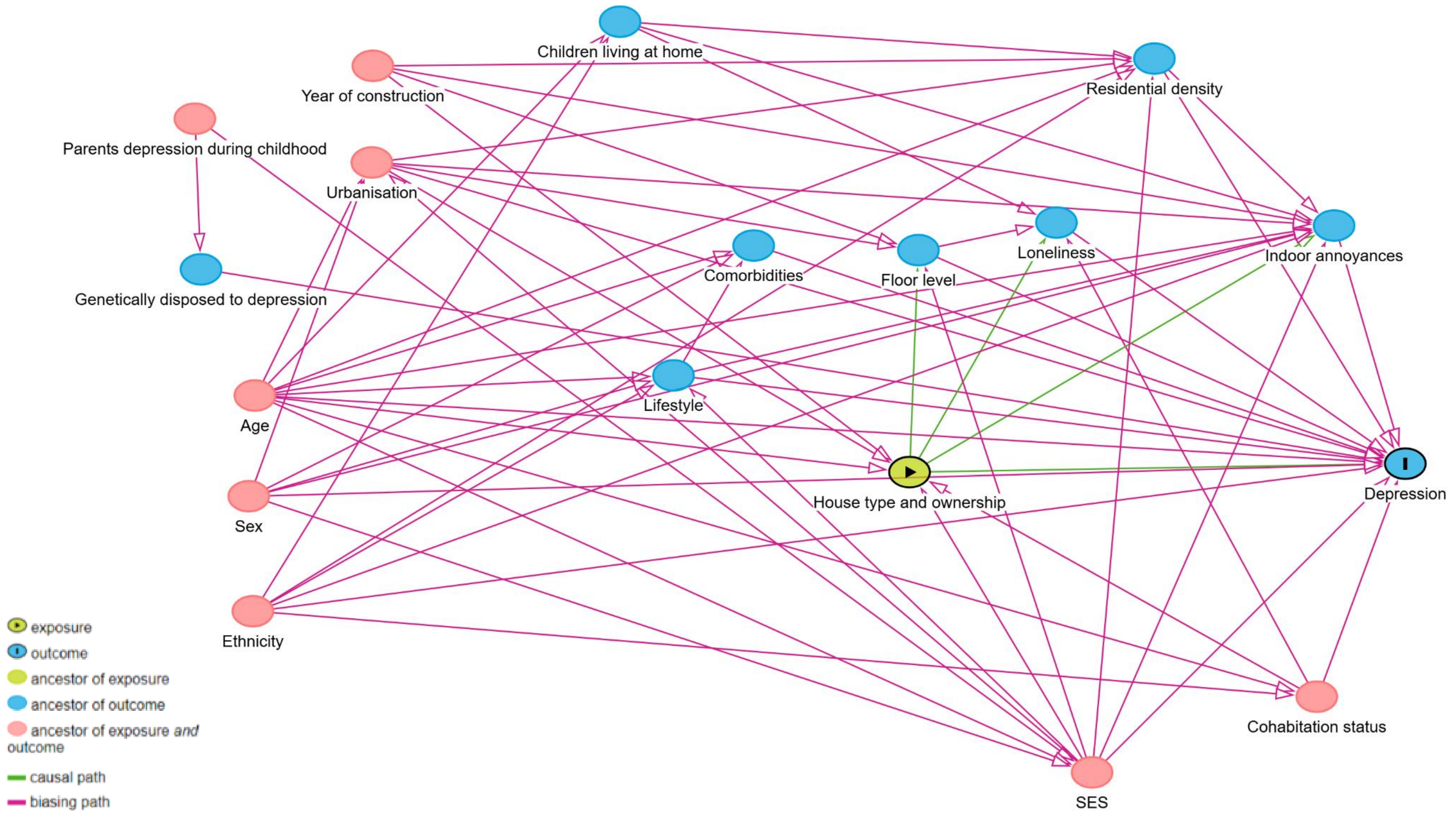


**Supplemental Figure 1.** Simplified Directed Acyclic Graph (DAG) highlighting variables of importance in the analysis of the association between housing type and depression.

**Supplemental Table 1.** Proportion mediated of housing type on depression for selected potential mediators [a]: number of perceived indoor annoyances due to either noise, thermal discomfort, low levels of light, or odour/stuffy air

| Potential mediator | Housing type | Proportion mediated, % (95% CI) [b, c] |
|---|---|---|
| **Noise annoyances [d]** | Owned terrace houses | 0.50 (-0.66, 4.08) |
| | Rented terrace houses | 2.43 (-2.42, 10.59) |
| | Rented apartments | **8.49 (1.83, 20.98)** |
| **Thermal annoyances [e]** | Owned terrace houses | -0.36 (-3.23, 0.91) |
| | Rented terrace houses | 4.25 (-0.65, 16.36) |
| | Rented apartments | **3.79 (1.03, 10.65)** |
| **Low light level annoyances [f]** | Owned terrace houses | 0.54 (-2.55, 7.62) |
| | Rented terrace houses | **2.40 (0.17, 7.85)** |
| | Rented apartments | **5.09 (0.83, 12.16)** |
| **Odour annoyances [g]** | Owned terrace houses | 0.17 (-0.96, 2.74) |
| | Rented terrace houses | **2.09 (0.02, 8.12)** |
| | Rented apartments | **2.82 (0.84, 7.87)** |

[a] Mediated effects were only estimated for those housing types that showed significant associations in the main analysis.
[b] Weighted for non-response.
[c] Exposure-outcome association and exposure-mediator association were adjusted for age, cohabitation status, educational level, urbanisation, year of housing construction, number of years lived in the residence at baseline, and calendar year. Except the mediation-analyses with low light-level annoyances as they were only adjusted for educational level.

Include following items (see [26,37,38]):
[d] Traffic noise, noise from installations, noise from neighbours, noise from nearby industry, infrasound or low-frequency sound, and vibration in building;
[e] Too low/high temperatures, draught, and draught along the floor;
[f] The dwelling was too dark;
[g] Odour or stuffy air, and shock from static electricity.

Owned detached houses were the reference group.
Bold text indicates statistical significance, $p<0.05$
CI, confidence interval.

**Supplemental Table 2.** Poisson regression of rates of depression among a sample of individuals with ≥21 points in the SF-36 mental health score at baseline (n=7,906) from the Danish Health and Morbidity Survey 2000

| **Housing type** | **N** (**7,906**) | **Incident depression** | | | | |
|---|---|---|---|---|---|---|
| | | **Number of events** (994) | **PYs at risk** (74,488) | **IR per 10,000 PY** (130) | **IRR** [a] **(95% CI)** | **Adjusted IRR** [a, b] **(95% CI)** |
| Owned detached houses | 4,178 | 567 | 48,057 | 118 | 1 (reference) | 1 (reference) |
| Owned terrace houses | 589 | 83 | 5,782 | 144 | 1.29 (1.02, 1.62) | 1.26 (0.98, 1.59) |
| Rented terrace houses | 653 | 81 | 4,127 | 196 | 1.68 (1.33, 2.10) | 1.35 (1.03, 1.74) |
| Rented apartments | 1,243 | 128 | 7,662 | 167 | 1.43 (1.20, 1.71) | 1.31 (1.04, 1.63) |
| Owned farms | 611 | 84 | 6,696 | 125 | 1.01 (0.78, 1.29) | 0.88 (0.64, 1.20) |
| Owned apartments and other housing types | 261 | 15 | 1,917 | 78 | 0.69 (0.41, 1.07) | 0.68 (0.40, 1.07) |
| Rented detached houses, farms and other housing types | 371 | 36 | 2,247 | 160 | 1.40 (0.97, 1.93) | 1.05 (0.71, 1.50) |

[a] Weighted for non-response.

[b] Adjusted for age, cohabitation status, educational level, urbanisation, year of housing construction**,** number of years lived in the residence at baseline, and calendar year.

N, number of individuals; PY, person years; IR, incidence rate; IRR, incidence rate ratio; CI, confidence interval.

**Supplemental Table 3.** Proportion mediated of housing type on depression for selected potential mediators[a] when including number of perceived indoor annoyances as a possible mediator among a sample of individuals with ≥21 points in the SF-36 mental health score at baseline

| Potential mediator | Housing type | Proportion mediated, % (95% CI) [b] |
|---|---|---|
| **Number of perceived indoor annoyances** [c] | Rented terrace houses | -2.41 (-20.35, 9.04) |
| | Rented apartments | 7.11 (-1.69, 35.24) |
| **Perceived loneliness** [d] | Rented terrace houses | 0.44 (-0.71, 4.10) |
| | Rented apartments | 2.51 (-1.26, 9.59) |

[a] Mediated effects were only estimated for those housing types that showed significant associations in the main analysis.
[b] Weighted for non-response.
[c] Exposure-outcome association and exposure-mediator association were adjusted for age, cohabitation status, educational level, urbanisation, year of housing construction, number of years lived in the residence at baseline, and calendar year.
[d] Exposure-outcome association and exposure-mediator association were adjusted for urbanisation and year of housing construction.
Owned detached houses were the reference group.
Bold text indicates statistical significance, $p<0.05$
CI, confidence interval.

**Supplemental Table 4.** Poisson regression of rates of depression among individuals with very good, good, and fair self-rated health at baseline (n=13,742) from the Danish Health and Morbidity Survey 2000

| **Housing type** | **N** (13,742) | **Incident depression** | | | | |
|---|---|---|---|---|---|---|
| | | **Number of events** (1,863) | **PYs at risk** (125,340) | **IR per 10,000 PY** (149) | **IRR [a] (95% CI)** | **Adjusted IRR [a, b] (95% CI)** |
| Owned detached houses | 6,885 | 997 | 76,063 | 131 | 1 (reference) | 1 (reference) |
| Owned terrace houses | 978 | 154 | 9,064 | 170 | 1.36 (1.15, 1.61) | 1.31 (1.09, 1.55) |
| Rented terrace houses | 1,252 | 168 | 7,715 | 218 | 1.69 (1.43, 1.97) | 1.27 (1.05, 1.53) |
| Rented apartments | 2,393 | 294 | 14,138 | 208 | 1.57 (1.39, 1.77) | 1.29 (1.10, 1.51) |
| Owned farms | 1,044 | 148 | 11,092 | 133 | 0.99 (0.82, 1.19) | 0.96 (0.76, 1.21) |
| Owned apartments and other housing types | 506 | 38 | 3,495 | 109 | 0.84 (0.61, 1.12) | 0.76 (0.54, 1.03) |
| Rented detached houses, farms, and other housing types | 684 | 64 | 3,772 | 170 | 1.35 (1.05, 1.72) | 0.99 (0.75, 1.30) |

[a] Weighted for non-response.

[b] Adjusted for age, cohabitation status, educational level, urbanisation, year of housing construction**,** number of years lived in the residence at baseline, and calendar year.

N, number of individuals; PY, person years; IR, incidence rate; IRR, incidence rate ratio; CI, confidence interval.

**Supplemental Table 5.** Proportion mediated of housing type on depression for selected potential mediators [a] among individuals with very good, good, and fair self-rated health at baseline

| Potential mediator | Housing type | Proportion mediated, % (95% CI) [b] |
|---|---|---|
| **Number of perceived indoor annoyances** [c] | Owned terrace houses | 0.31 (-0.44, 2.70) |
| | Rented terrace houses | 6.78 (-0.72, 38.60) |
| | Rented apartments | **11.80 (5.21, 32.45)** |
| **Perceived loneliness** [d] | Owned terrace houses | 2.34 (-2.43, 11.80) |
| | Rented terrace houses | N/A |
| | Rented apartments | **5.81 (2.11, 12.52)** |

[a] Mediated effects were only estimated for those housing types that showed significant associations in the main analysis.
[b] Weighted for non-response.
[c] Exposure-outcome association and exposure-mediator association were adjusted for age, cohabitation status, educational level, urbanisation, year of housing construction, number of years lived in the residence at baseline, and calendar year.
[d] Exposure-outcome association and exposure-mediator association were adjusted for urbanisation and year of housing construction.
Owned detached houses were the reference group.
Bold text indicates statistical significance, $p<0.05$
CI, confidence interval.

**Supplemental Table 6.** Poisson regression of rates of depression among a sample of individuals ≥30 years old (n=11,993) from the Danish Health and Morbidity Survey 2000

| **Housing type** | **N** (11,993) | **Incident depression** | | | | |
|---|---|---|---|---|---|---|
| | | **Number of events** (1,968) | **PYs at risk** (121,732) | **IR per 10,000 PY** (162) | **IRR** [a] **(95% CI)** | **Adjusted IRR** [a, b] **(95% CI)** |
| Owned detached houses | 6354 | 1063 | 74891 | 142 | 1 (reference) | 1 (reference) |
| Owned terrace houses | 885 | 152 | 8857 | 172 | 1.27 (1.07, 1.50) | 1.21 (1.01, 1.43) |
| Rented terrace houses | 1021 | 185 | 7386 | 250 | 1.78 (1.53, 2.07) | 1.29 (1.08, 1.54) |
| Rented apartments | 1821 | 306 | 12945 | 236 | 1.66 (1.47, 1.86) | 1.28 (1.10, 1.49) |
| Owned farms | 952 | 149 | 10860 | 137 | 0.95 (0.78, 1.14) | 0.88 (0.70, 1.10) |
| Owned apartments and other housing types | 407 | 39 | 3192 | 122 | 0.89 (0.65, 1.18) | 0.73 (0.52, 0.99) |
| Rented detached houses, farms and other housing types | 553 | 74 | 3601 | 205 | 1.57 (1.24, 1.95) | 1.13 (0.87, 1.44) |

[a] Weighted for non-response.

[b] Adjusted for age, cohabitation status, educational level, urbanisation, year of housing construction, number of years lived in the residence at baseline, and calendar year.

N, number of individuals; PY, person years; IR, incidence rate; IRR, incidence rate ratio; CI, confidence interval.

**Supplemental Table 7.** Proportion mediated of housing type on depression for selected potential mediators [a] among a sample of individuals ≥30 years old

| Potential mediator | Housing type | Proportion mediated, % (95% CI) [b] |
|---|---|---|
| **Number of perceived indoor annoyances [c]** | Owned terrace houses | 0.24 (-1.31, 3.18) |
| | Rented terrace houses | 5.53 (-0.32, 27.57) |
| | Rented apartments | **10.64 (4.49, 42.09)** |
| | Owned apartments and other housing types | -5.71 (-47.28, 47.79) |
| **Perceived loneliness [d]** | Owned terrace houses | 3.37 (-3.82, 19.79) |
| | Rented terrace houses | N/A |
| | Rented apartments | **9.24 (4.35, 17.85)** |
| | Owned apartments and other housing types | -14.41 (-603.14, 448.70) |

[a] Mediated effects were only estimated for those housing types that showed significant associations in the main analysis.
[b] Weighted for non-response.
[c] Exposure-outcome association and exposure-mediator association were adjusted for age, cohabitation status, educational level, urbanisation, year of housing construction, number of years lived in the residence at baseline, and calendar year.
[d] Exposure-outcome association and exposure-mediator association were adjusted for urbanisation and year of housing construction.
Owned detached houses were the reference group.
Bold text indicates statistical significance, p<0.05
CI, confidence interval.

**Supplemental Table 8.** Table of perceived indoor annoyances and perceived loneliness

| | | **Number of perceived annoyances N (% i columns)** | | | |
|---|---|---|---|---|---|
| | | **0 annoyances** | **1 annoyance** | **2 annoyances** | **≥3 annoyances** |
| **Perceived loneliness** | **Frequently** | 208 (1.97) | 97 (4.03) | 51 (5.76) | 31 (6.53) |
| | **Occasionally** | 959 (9.07) | 374 (15.53) | 167 (18.85) | 116 (24.42) |
| | **Rarely** | 1016 (9.61) | 455 (18.89) | 162 (18.28) | 103 (21.68) |
| | **Never** | 8390 (79.35) | 1,483 (61.56) | 506 (57.11) | 225 (47.37) |

N=14,343; Chi-squared test showed p-value <0.0001.
